# The Role of Ionic Liquids in Textile Processes: A Comprehensive Review

**DOI:** 10.3390/molecules30020353

**Published:** 2025-01-16

**Authors:** Anastasia Anceschi, Claudia Riccardi, Alessia Patrucco

**Affiliations:** 1 CNR-STIIMA, Italian National Research Council, Institute of Intelligent Industrial Technologies and Systems for Advanced Manufacturing, 13900 Biella, Italy; anastasia.anceschi@stiima.cnr.it; 2 Dipartimento di Fisica, Università di Milano-Bicocca, Piazza della Scienza 3, 20126 Milano, Italy; claudia.riccardi@unimib.it

**Keywords:** textile, ionic liquids, sustainability

## Abstract

Thanks to their unique physicochemical properties, ionic liquids (ILs) have moved from niche academic interest to critical components in various industrial applications. The textile industry, facing significant environmental and economic pressures, has begun to explore ILs as sustainable alternatives to traditional solvents and chemicals. This review summarizes research on the use of ILs in various textile processes, including dyeing, finishing, and fiber recycling, where their high thermal stability, tunable solubility, and low volatility are exploited to reduce resource consumption and environmental impact. The discussion also extends to the integration of ILs in textile waste recycling, highlighting innovative approaches to fiber dissolution and regeneration aimed at circular economy goals. Despite these advances, challenges such as high production costs and scalability remain barriers to the widespread adoption of ILs in the textile sector. Addressing these barriers through continued research and development is essential to fully realize the potential of ILs for sustainable transformation in textiles.

## 1. Introduction

**Figure 1:**
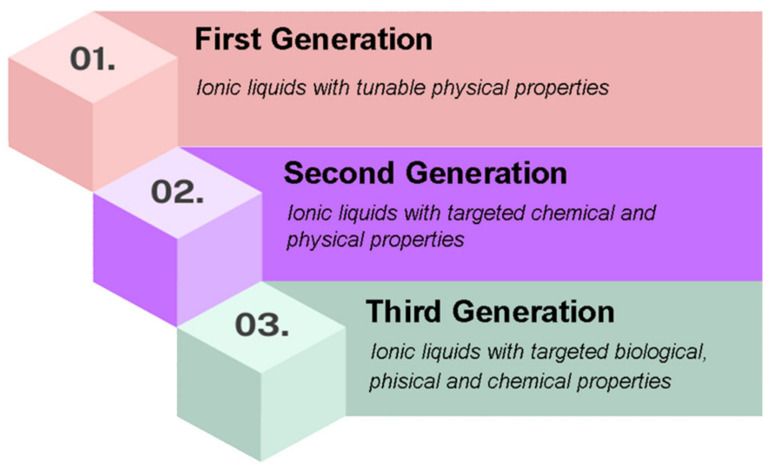
Ionic liquid generations.

Twenty years ago, research into ionic liquids was a small area of academic interest, with only a limited number of scientists investigating salts with melting points close to room temperature. Today, there are over 125,000 scientific publications and 17,000 patent applications in the field of ionic liquids [1]. The first ionic liquids were reported in 1877 and were called “red oil”. They were obtained as a separate phase in Friedel–Crafts reactions [2,3]. The substance was subsequently identified as a chloroaluminate with an alkylated aromatic cyclic cation [4]. Other early ionic liquids were identified at the turn of the century, including alkylpicolinium halides and quaternary anilinium salts [5,6]. These substances were also referred to as “intractable oils”, indicating that a new phenomenon was emerging. From these early compounds, the history of these innovative compounds continued until 1943, when the scientist Barrer coined the first name to define them: ionic liquids [7]. The history of ionic liquids can be described in terms of “generations” [8]. Three distinct generations of ionic liquids can be distinguished, as summarized in Figure 1.

- First generation

The first known ionic liquid, ethylammonium nitrate, was identified by Walden [9]. However, the scientific community has identified a divergence in the attribution of the initial synthesis of ionic liquids (ILs). Some experts ascribe the pioneering achievement of IL synthesis, specifically to Gabriel and Weiner, citing the 1888 synthesis of ethanolammonium nitrate [10]. This discrepancy stems from the reported melting point of the substance. While Walden’s compound was found to have a melting point of 13–14 °C, Gabriel and Weiner’s formulation possessed a melting point of over room temperature (around 50 °C). These findings signify the inception of the initial generation of ILs [8].

Subsequently, a number of other ionic liquids were synthesized using a variety of anions and cations. In particular, the cations, including alkylpyridinium, alkylimidazolium, and dialkylimidazolium, have been the most extensively studied. For anions, chloroaluminate and metal halides have been the most widely used. This first generation of ionic liquids showed high reactivity with water and air, which required the use of inert conditions, making them unsuitable for use in open conditions [2].

- Second generation

To overcome the problem of high reactivity with water exhibited by the first generation of ionic liquids, the traditional anions were replaced by chloroaluminate anions, including Cl^−^, Br^−^, I^−^, PF_6_^−^, BF_4_^−^, and C_6_H_5_COO^−^, while the cations were replaced by ammonium and phosphonium-based cations, in addition to alkylpyridinium, alkylimidazolium, and dialkylimidazolium [11]. This second generation of ionic liquids has remarkable properties such as low melting point, low viscosity, and high solubility. This new class of ionic liquids has been rapidly adopted in the field of biocatalysis, leading to a significant increase in research activity in this area [12]. Despite the promising potential of the second generation of ionic liquids, they were found to have toxic properties and were associated with a significant financial burden in terms of production costs [13].

- Third generation

In order to improve environmental sustainability and reduce the financial burden associated with the synthesis of ionic liquids, the cations have predominantly been replaced by choline. However, for cations, a variety of alternative species were investigated, including amino acids, alkylphosphates, alkylsulfates, bis(trifluoromethanesulfonyl)amide (TFSI) [(CF_3_ SO_2_)_2_ N^−^], and sugars [14]. This third generation of ionic liquids exhibits remarkable properties that can be tailored based on the anions or cations used. In addition, this third generation of ionic liquids comprises a novel class of solvent system called deep eutectic solvents (DES) [15]. These substances are essentially mixtures of salts, including choline chloride, alcohols, amides, carboxylic acids, amines, and urea.

The third generation of ionic liquids has excellent properties that make them suitable for exploitation for commercial use [14]. The potential use of ionic liquids has been explored in various fields, such as hydrogenation, oxidation, Diels-Alder reaction, electrochemical reactions, biocatalysis, nanomaterial synthesis, hydrogen storage, liquid mirror production, and separation processes [16,17,18,19,20,21,22,23,24]. Despite the potential benefits, the use of ionic liquids in some sectors, such as the textile industry, remains relatively limited and restricted to a narrow range of applications [25].

The textile industry is an important sector with a significant economic and environmental impact. Conventional textile production processes often involve the extensive use of water, energy, and chemicals, ultimately leading to environmental pollution and depletion of natural resources [26]. Ionic liquids, with distinctive properties such as low vapor pressure, high thermal stability, and tunable solubility, offer a compelling prospect as replacements for a range of textile applications. Accordingly, this review aims to assess the current state of research into the use of ionic liquids in the textile industry, with particular emphasis on their applications, benefits, challenges, and future developments.

## 2. Overview of Ionic Liquids

### 2.1. Structure of Ionic Liquids

**Figure 2:**
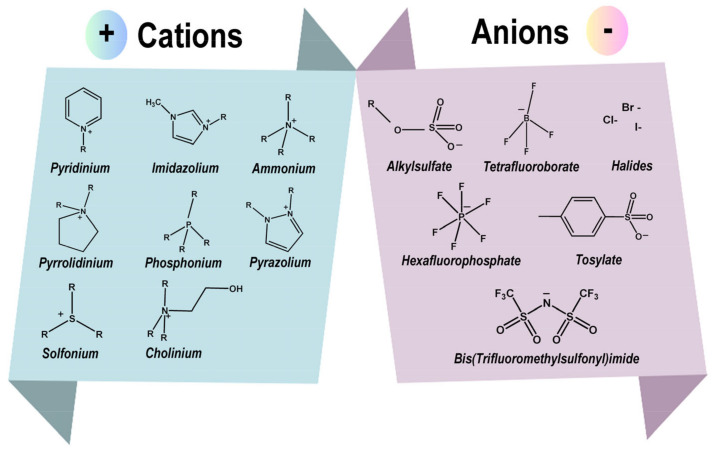
Common cations and anions that are present in ionic liquids.

The exact definition of ionic liquids remains controversial. The definition of ionic liquids has evolved over time. Historically, the term “room temperature ionic liquids” (RTILs) was used to refer to ionic liquids that are liquid at 0.1 MPa and at or below 100 °C, the boiling point of water. However, this definition has become less common as the field has expanded to include ionic liquids with a broader range of melting points and applications. In any case, ionic liquids are typically composed of asymmetric and univalent ions, which prevent tight crystal packing and consequently lower the melting point [27]. Therefore, the choice of cation and anion is an important consideration in the design of an ionic liquid. There are currently nearly 1000 ionic liquids documented in the literature, and the main cations and anions are shown in Figure 2.

The choice of cations appears to be limited in many organic chemistry groups, but they can be divided into two categories. The first category includes phosphonium, sulphonium, chloronium, and ammonium ions, which are classified as acyclic. The second class is classified as the cyclic group and includes molecules such as pyridinium, imidazolium, pyrazolium, and pyrrolidium ions [28]. For anions, the range of possibilities is wider, and the most commonly used anions are halides such as chloride and bromide, alkyl sulphate derivatives, thiocyanate, triflate, tosylate, and fluorophosphate [24]. The possible combinations of cations and anions are limitless, resulting in a wide range of ionic liquids that can be used in a variety of applications.

### 2.2. Properties of Ionic Liquids

**Figure 3:**
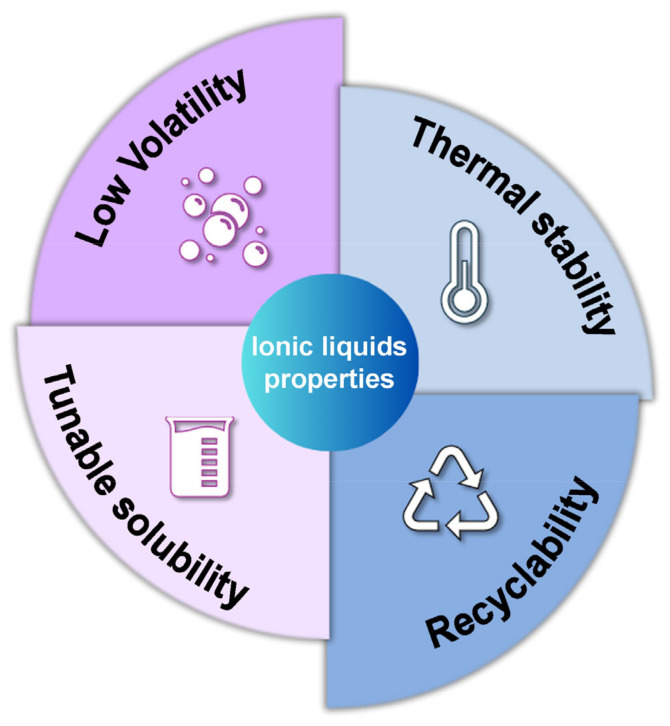
Common properties of ionic liquids.

Due to their unique properties, ionic liquids are attracting increasing interest as potential replacements for existing organic solvents. Among the most important properties of ionic liquids are the following. A brief illustration of the properties of ionic liquids is shown in Figure 3.

- Low Volatility

The main advantage of ionic liquids is that they have negligible vapor pressure. This is a significant advantage over conventional organic solvents. This allows products to be removed by distillation without the risk of further contamination, reducing the risk of air pollution and occupational hazards [27].

- High Thermal Stability

Ionic liquids can withstand high temperatures, a significant advantage over traditional solvents. Ionic liquids have remarkable stability even at elevated temperatures and nonflammable properties, making them a viable alternative to traditional solvents in applications involving high temperatures and the possibility of explosions [29]. This includes the textile industry, where ionic liquids offer a safer alternative for use in various processes [30].

- Tunable Solubility

It is possible to alter the solvating properties of ionic liquids through appropriate modifications. Specifically, the physical, chemical, or biological properties of ionic liquids can be adjusted or modified by changing the ratio of cations to anions or by incorporating specific functionalities into the cations or anions. Finally, the combination of two or more ionic liquids can be used to achieve desired properties. As a result, ionic liquids offer a distinct advantage over conventional solvents in their ability to dissolve a wide range of organic and inorganic compounds [24].

- Recyclability

Due to their chemical stability, ionic liquids can be recycled and reused as solvents with excellent recovery rates. In addition, the use of ionic liquids as reaction media can facilitate the recycling and recovery of catalysts in chemical processes. Therefore, the use of ionic liquids can effectively reduce the generation of waste [31].

Summarizing, the excellent properties of ionic liquids are intrinsically linked to their unique structure–property relationships. Ionic liquids are composed entirely of ions, typically a bulky, asymmetrical cation paired with a smaller anion. This ionic composition disrupts lattice packing, leading to lower melting points and high fluidity at room temperature. Furthermore, the electrostatic interactions between ions confer high thermal stability, while their negligible vapor pressure stems from the strong Coulombic forces that prevent evaporation under normal conditions [32]. The tunable solubility of ILs is a particularly noteworthy property. By altering the cation or anion structure—such as incorporating functional groups (e.g., hydroxyl, alkyl, or aromatic groups)—researchers can modulate solvation properties, polarity, and hydrophilicity/hydrophobicity. For example, imidazolium-based ILs with longer alkyl chains exhibit enhanced solubility for nonpolar solutes, while ILs with functionalized anions can improve compatibility with polar compounds [33]. This structure–property relationship enables the customization of ionic liquids for specific applications, such as dye solubilization, fiber modification, or environmental remediation, making them versatile tools for the textile industry and beyond [34].

## 3. Applications of Ionic Liquids in Textile Processes

**Figure 4:**
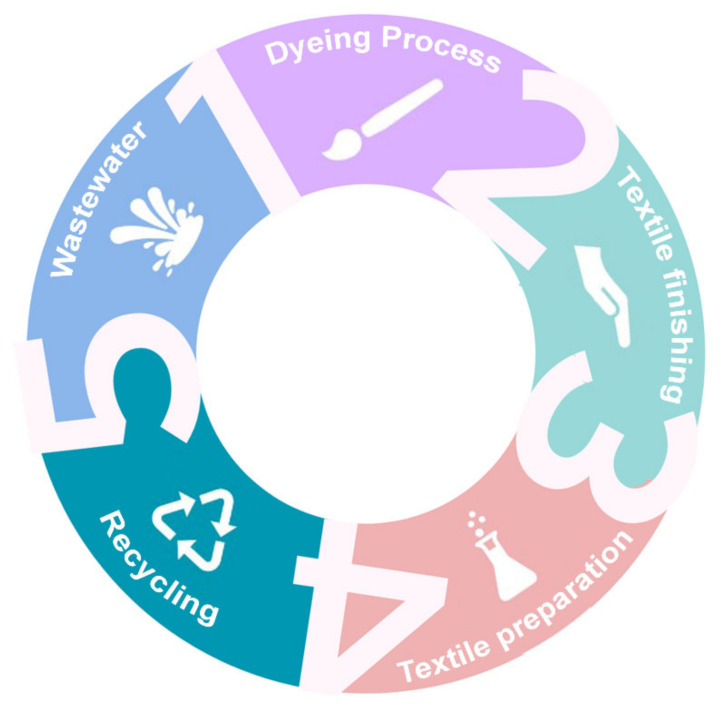
Sectors of the textile industry where ionic liquids are used.

After a brief overview of ionic liquids and their properties, this section will examine the applications of ionic liquids in the textile industry. The textile industry is a major contributor to global pollution, with extensive use of water, energy, and chemicals. Conventional solvents used in textile processing are often toxic and nonbiodegradable, posing serious environmental risks. In light of these considerations, there has been a recent shift toward research into more sustainable alternatives. In this context, it is interesting to examine the applications of ionic liquids, which are increasingly being studied and used in the textile sector [35]. A summary of the textile sectors where the use of ionic liquids is beginning to attract attention is shown in Figure 4.

### 3.1. Dyeing Processes

Dyeing is a key step in the textile manufacturing process. However, traditional dyeing techniques rely on extensive water and energy consumption, resulting in significant waste. Ionic liquids can offer an alternative solution, as they have been shown to provide several benefits when used in dyeing processes [35]. For example, they facilitate dye solubility as they are able to dissolve a wide range of dyes, including those that are poorly soluble in water. They also improve the durability of the dye, reducing the likelihood of fading over time and facilitating the dyeing process, reducing the time and energy required for production. From an economic and environmental perspective, ionic liquids can be a viable alternative to water as a solvent, significantly reducing water consumption and wastewater generation [6,35,36]. Their potential applications in the textile dyeing process are outlined in Table 1.

The underlying mechanisms by which ionic liquids (ILs) enhance textile dyeing processes are multifaceted. In particular, ionic liquids are used for:

- Dye Solubility: Ionic liquids exhibit remarkable solvation properties attributable to their ionic composition and tunable polarity. Imidazolium-based ionic liquids, for instance, effectively dissolve a wide range of dyes, including those sparingly soluble in water, by disrupting dye aggregates and enhancing molecular dispersion [45]. This results in higher dye solubility, which is critical for achieving uniform and vibrant coloration.
- Fiber Modification: Ionic liquids can chemically modify fiber surfaces to enhance dye absorption. For example, when used on polyamide fibers, ionic liquids can generate positive charges on the fiber surface, strengthening electrostatic interactions with anionic dyes [38]. Similarly, for natural fibers like wool and silk, ionic liquids partially disrupt the keratin or cellulose network, allowing deeper penetration of dye molecules [39].
- Temperature and Energy Efficiency: Ionic liquids facilitate dyeing at reduced temperatures, minimizing thermal degradation of both dye and fiber. This property not only conserves energy but also supports the preservation of fabric integrity, particularly for heat-sensitive textiles [35].

An interesting methodology that explores the use of ionic liquids in the textile dyeing process comprises two phases. In the initial phase, the textile fibers are subjected to a pretreatment process involving the use of ionic liquids. This is followed by a second phase, in which the fibers are dyed using a conventional methodology. This procedure was initially proposed by Yuan and colleagues, who investigated the potential of 1-butyl-3-methylimidazolium chloride in the dyeing of wool fibers [37]. It was established that these ionic liquids are capable of effectively damaging the external cuticles of wool fibers, facilitating a greater penetration of dyes. These notable outcomes were additionally corroborated by Kantouch et al. [39]. In their study, the impact of ionic liquids on the dyeability of wool and silk was investigated. Notably, the employment of ionic liquids appears to facilitate the dyeing of wool and silk fabrics with acid and reactive dyes at relatively low temperatures. Other noteworthy results were reported by Dong et al. [38]. The objective of the study was to investigate the impact of four distinct 1-alkyl-3-methylimidazolium bromine ionic liquids on the adsorption capacity of nylon 6.6 fibers. It was observed that the amount of dye adsorbed was directly proportional to the concentration and the length of the alkyl groups of the ionic liquids when the pH was maintained at 5 and 7. This phenomenon can be attributed to the ability of ionic liquids to generate positive charges on the surface of nylon 6.6 fibers, thereby enhancing the ability of nylon 6.6 fibers to absorb the dye.

**Figure 5:**
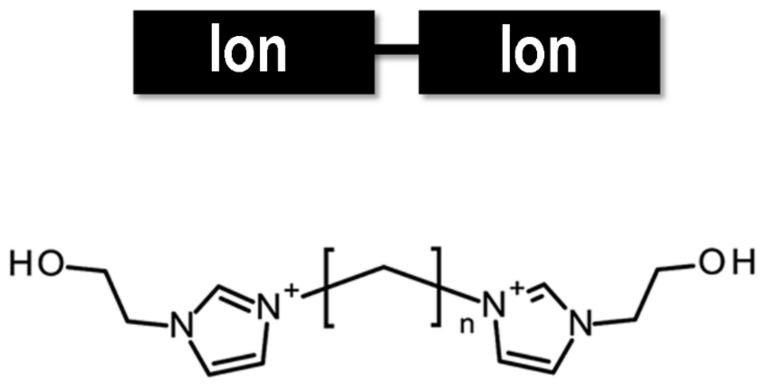
Schematic representation and example of gemini ionic liquids.

Recently, a new class of ionic liquids has attracted considerable interest in the field of fiber dyeing: Gemini ionic liquids. They typically consist of two ionic groups linked by a spacer [46]. A generic representation is reported in Figure 5.

Nevertheless, the potential for their application in dyeing has been investigated by Zhuang and colleagues [47]. In particular, a gemini dicationic imidazolium ionic liquid has been synthesized for the pretreatment of ramie fibers to increase their dye uptake capacity. This study examined the impact of treatment conditions, specifically temperature and duration time, on the color strength of ramie fibers. The ramie fibers were subjected to a dyeing process using CI Reactive Yellow 4, with a concentration of 0.1%, at a bath ratio of 1:30. The study revealed a significant correlation between the treatment temperature and the K/S value of the fibers. It was observed that as the temperature increased from 20 °C to 80 °C, the K/S value exhibited a marked increase, reaching a maximum of 16.7 at 100 °C. Conversely, the effect of treating time on K/S value was studied by varying reacting times from 10 min to 60 min. In this case, the K/S value increased to 15.6 as the reaction time was extended. However, after 30 min, the K/S value stabilized.

Another notable application of ionic liquids is as auxiliaries in dyeing baths [36]. The experimental results show that the use of these ionic liquids results in comparable dye uptake and fixation rates to those achieved with conventional inorganic salts, thereby establishing their viability as an alternative to the dominant textile auxiliaries. Similarly, Cheng and colleagues observed comparable results [48]. In this case, ionic liquids were used to replace sodium chloride in a Reactive Red B dye bath. The results showed that this ionic liquid acts as an accelerator in silk dyeing, facilitating the rate of dye uptake. In particular, the results obtained from this study indicated that the rate of dye uptake was approximately 85.4%, and the K/S value was 11.2. In fact, the proposed ionic liquids are capable of interacting with acidic groups on the silk surface, thereby enhancing the electrostatic interaction between the dyes and the silk.

Another interesting research study has shown that ionic liquids can act as efficient dye retardants. In particular, imidazole-based ionic liquids were found to be effective in dyeing acrylic fibers [43]. Furthermore, Bianchini et al. have demonstrated the feasibility of wool, polyester, and cotton dyeing in an open vessel using dispersed dyes and appropriate ionic liquids [35]. In particular, the use of 1-(2-hydroxyethyl)-3-methylimidazolium chloride resulted in excellent dyeing of wool and polyester with good colorfastness. In other studies, the potential of ionic liquids as solvents in the dyeing of cellulosic fibers, proteins, and fiber blends was investigated [40,42].

Another methodology for the use of ionic liquids in textile dyeing involves their employment as auxiliaries within the dyeing bath. A Chinese patent details the synthesis of quaternary ammonium salt ionic liquids and their application in the dyeing of cotton with triazine reactive dyes. In this case, the ionic liquid was used with a concentration of 10–20 g/L to replace traditional inorganic salts, resulting in a dye uptake and fixation rate that was comparable to that of traditional dyeing methods. The dyeing process was conducted at a temperature of 60 °C, with the pH of the dyeing bath being maintained within the range of 8–11 [49].

Furthermore, Barros and colleagues investigated the potential application of ionic liquids as dye auxiliaries in aqueous dyeing procedures [36]. In their study, they examined various types of fabrics in conjunction with a polyfunctional reactive dye and protic ionic liquids. The researchers examined a diverse array of fibers, encompassing diacetate, cotton, polyester, polyamide, acrylic, and wool. The K/S value exhibited a range consistent with the utilized ionic liquids. The reported K/S values were found to be approximately 1.5 for diacetate, 0.60 for cotton, 0.5 for polyamide, 0.50 for polyester, and 0.8 and 0.5 for acrylic and wool, respectively.

Recently, the dyeing of m-aramid fibers using ionic liquids has also been investigated. Indeed, the established dyeing process for aramid fibers fails to meet the contemporary benchmarks for occupational safety and environmental protection. Consequently, novel methodologies must be developed to address these shortcomings. In this approach, Opwis et al. discovered an effective method for dyeing m-aramid fibers utilizing 1-ethyl-3-methylimidazolium ethyl sulfate with acid dyestuffs. Specifically, they developed a process for dyeing m-aramide fibers without the use of hazardous carriers, achieving excellent coloration and outstanding wash, rub, and light fastness [41].

Compared to conventional water-based dyeing methods, ionic liquids offer several distinctive advantages. Traditional methods often require large amounts of water as a solvent, which leads to high water consumption and significant effluent generation containing unbound dyes and auxiliaries [41,50]. Ionic liquids, on the other hand, minimize water usage by serving as the primary medium for dye solubility. Furthermore, the enhanced dye uptake facilitated by ionic liquids reduces dye wastage and effluent toxicity. Their ability to operate effectively at lower temperatures also contrasts with conventional high-temperature dye baths, resulting in notable energy savings [51,52].

Other notable research into ionic liquids involves their use in textile printing. In this case, ionic liquids have been used as a component in the formulation of aqueous ink compositions for inkjet printing, with the aim of replacing hazardous solvents, preventing dye precipitation and providing good heat stability [53]. In a more recent study, methylimidazolium-based ionic liquids were investigated for their potential use in the burn-out printing of polyester/viscose blend fabrics [54]. Although the method investigated appears to be efficient and promising, it has not yet been demonstrated that it can completely replace the conventional technique.

### 3.2. Fiber Preparation and Textile Finishing

**Figure 6:**
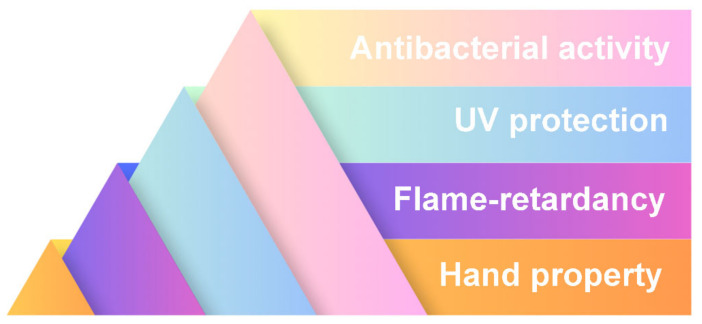
Example of applications of ionic liquids in textile finishing.

Textile finishing is a process used in the manufacture of fibers, fabrics, or garments to impart new and desired functional properties. Typically, the finishing process includes treatments designed to improve the softness, durability, wrinkle resistance, water repellency, or flame retardancy of the material. The finishing process plays a key role in the textile manufacturing process, determining the final properties and quality of the fabric and ensuring the suitability of the finished product for its intended use. Ionic liquids have shown promise as textile finishing agents, as summarized in Figure 6.

The application of ionic liquids in textile finishing extends beyond physical modification to include chemical interactions that impart functional properties. For example:

- Antibacterial properties: Imidazolium-based ionic liquids chemically bond with keratin in wool, forming stable complexes that exhibit durable antibacterial effects against both Gram-positive and Gram-negative bacteria, even after repeated washing [55].
- UV resistance and flame retardancy: Ionic liquids facilitate the incorporation of agents such as nano-zinc oxide or phosphonium cations [56,57]. This results in textiles with enhanced UV protection and improved flame-retardant properties without requiring additional chemical agents.
- Surface modification: Ionic liquids enable surface-level transformations of synthetic fibers, such as imparting a cotton-like texture to polyester through deposition of cellulosic material [58]. These interactions are facilitated by the unique solvating and catalytic properties of ionic liquids, making them versatile tools for advanced textile finishing.

The use of ionic liquids in textile finishing processes is typically associated with the imparting of antibacterial and antimicrobial properties. The various applications are summarized in Table 2:

For example, Kantouch and colleagues investigated the effect of treating wool fabric with two imidazolium-based ionic liquids in order to impart long-lasting antimicrobial properties [59]. It was found that pretreatment of wool was necessary to induce structural changes that facilitated the binding of ionic liquids to the keratin structure. This process resulted in a stable antibacterial effect against *Escherichia coli*. However, the antimicrobial activity of wool fabric treated with ionic liquid at different temperatures is a subject of considerable variation. Wool fabric treated with ionic liquid at 30 °C, for instance, is not durable for washing and is reduced by about 50% after 10 washing cycles and entirely eliminated after 20 washing cycles. Conversely, wool fabric treated with ionic liquid at 80 °C exhibited persistent antibacterial properties, maintaining its efficacy even after 20 washing cycles. In a similar study, the potential antibacterial effect of ionic liquids on viscose was investigated [60]. In this case, the viscose fabric was first treated with anionic agents and then with ionic liquids. The treated viscose exhibits remarkable antibacterial resistance to both Gram-positive and Gram-negative bacteria, even after 10 wash cycles. In addition to antibacterial properties, ionic liquids can be used to impart other desirable properties, such as UV resistance and flame retardancy. For example, Arputharaj et al. applied nano-zinc oxide to cotton fabric using 1-butyl-3-methyl imidazolium chloride [63]. The treated cotton fabric exhibited remarkable bacteriostatic activity against both Gram-positive and Gram-negative bacteria, as well as excellent UV protection and a certain durability.

In addition, the presence of ammonium, phosphonium, and sulphonium cations in a number of ionic liquids prompted research into their potential as flame-retardant coatings for textile materials. As numerous studies have shown, this new class of flame retardants based on ionic liquids offers several advantages [57,62]. These include the absence of hazardous compounds, minimal volatility, and thermal stability. For example, Bentis and colleagues developed a cotton fabric treatment using methylimidazolium and pyridinium cations combined with anions including Cl^−^, PF_6_^−^, (CF_3_SO_2_)_2_N^−^, BF_4_^−^, and CH_3_CO_2_^−^ in a sol-gel process. The LOI test showed a superior LOI value of 25% for the treated cotton fabrics compared to the untreated fabrics (LOI = 20%), suggesting that the ionic liquid-based treatment has improved the protective capacity of the fabric against degradation [61]. Finally, Opwis et al. presented an intriguing application of ionic liquids [56], their paper describes the development of a novel finishing process for polyester fibers. Specifically, the process involves the deposition of cellulosic materials onto polyester fibers using ionic liquids, which creates a cotton surface on the synthetic fibers, imparting the tactile quality associated with the natural fiber.

The initial investigation into the potential applications of ionic liquids was conducted on the use of 1-ethyl-3-methylimidazolium acetate and 1-butyl-3-methylimidazolium chloride on the felting resistance of wool [55]. This study considers a number of factors, including temperature and time, the yellowing index, tenacity, and elongation at the break of the treated wool fibers. In a similar study, Yuan et al. demonstrated that treating wool with 1-butyl-3-methylimidazolium chloride significantly alters its surface state [64]. In another interesting study, the use of two ionic liquids for the degumming of silk was explored. In this case, the 1-butyl-3-methylimidazolium chloride and 1-butyl-3-methylimidazolium hydrogen sulfate were successfully applied as a green solvent for the silk degumming process, resulting in better weight loss and absorbency than the conventional hydrogen peroxide process [65]. An additional intriguing approach is the alkaline hydrolysis of polyester, which has the potential to impart hydrophilicity, a smooth surface, and brightness [58]. In this study, polyester was treated with methanolic solutions of sodium hydroxide in the presence of a quaternary ammonium compound, cetyltrimethylammonium bromide, and 1-butyl-3-methylimidazolium chloride. The process yielded promising results in comparison to the conventional hydrolysis process with sodium hydroxide, indicating its potential as an alternative method.

In general, in comparison to conventional finishing techniques, which often rely on a combination of water-based processes and chemical agents, ionic liquids stand out due to their versatility and efficiency. For instance, conventional flame retardants or antibacterial finishes frequently involve the use of volatile or toxic chemicals that can have adverse environmental impacts. Ionic liquids, by contrast, achieve similar functionalization with minimal emissions and better recyclability [66]. Additionally, their low volatility and chemical stability make them safer alternatives for certain high-temperature processes, such as flame-retardant treatments, compared to conventional solvent-based systems.

### 3.3. Recycling of Textile Waste

Textile waste is a major environmental challenge, with millions of tons of discarded fabrics ending up in landfill every year [67]. The rapid growth of the fast-moving fashion industry has accelerated this problem, as clothing is often produced cheaply, worn for a short time, and discarded quickly. This waste is not only the result of consumer behavior but also includes significant amounts of preconsumer waste generated during manufacturing, such as fabric scraps, offcuts and unsold stock [68,69]. In addition, most textiles are made of synthetic fibers such as polyester, which are difficult to treat. The high volume of textile waste contributes to the depletion of natural resources, increased greenhouse gas emissions, and severe contamination of water and soil, so there is an urgent need to develop sustainable alternatives for the production, consumption, and disposal of textiles. In this context, ionic liquids offer a promising solution to enable more sustainable processing and recycling methods within the textile industry. Due to their intrinsic properties, ionic liquids can effectively dissolve and separate different components of textile materials. As shown in Table 3, there have been some developments in this area of research.

In 2002, Swatloski et al. carried out the first study on the dissolution of cellulose in ionic liquids. These solvents show considerable potential for cellulose processing and the production of regenerated cellulose fibers such as rayon [78]. A mechanism for dissolving cellulose in ionic liquids was then proposed, involving a direct chemical reaction that disrupts the hydrogen bonding network of cotton fibers [79]. Nevertheless, Bentivoglio and coworkers are investigating the use of a number of ionic liquids, including 1-butyl-3-methylimidazolium chloride and 1-allyl-3-methylimidazolium chloride, as potential replacements for N-methylmorpholine N-oxide in the commercial production of Lyocell fibers [70]. In addition, Assadi et al. present a novel chemical recycling process for cotton waste, which enables the production of high-quality virgin textile fibers of significantly higher quality than those produced by the mechanical recycling processes currently in use [57]. In this case, postconsumer cotton waste was completely dissolved in 1,5-diazabicyclo[4.3.0]non-5-enium acetate to produce continuous filaments with high tenacity and good modulus, exceeding the mechanical properties of virgin cotton fibers [57]. More recently, the potential use of ionic liquids for the separation and recovery of textile blends has been investigated. For example, Haslinger et al. investigated the potential of a superbase-based ionic liquid for the separation of cotton and polyester [72]. The selective dissolution of the cotton allowed the removal of the polyester, and the solution could then be used to dry-jet, wet-spin, textile-grade cellulose fibers with good tenacity and elongation comparable to those of commercial lyocell fibers. Another interesting approach explored was the use of 1-allyl-3-methylimidazolium chloride to separate and recover cotton from cotton/polyester blends [73]. In their work, cotton waste was directly dissolved and coagulated in water, demonstrating the potential for this process to be developed into a highly promising technology with significant industrialization potential. Another interesting study investigated the potential of 1-allyl-3-methylimidazole chloride in combination with DMSO to dissolve cotton from poly/cotton waste [74]. In their work, the dissolution temperature, time, and performance were subjected to comprehensive investigation. The combination of 1-allyl-3-methylimidazole chloride and DMSO yielded favorable results, but the 1-ethyl-3-methylimidazolium diethyl phosphate DMSO system demonstrated superior performance in terms of separation. This ionic liquid exhibited a viscosity of 0.1 Pa·s, which was advantageous for the separation of polyester from the cellulose solution. Additionally, Sun et al. investigated the potential for the separation and regeneration of cotton/polyester and wool/polyester blended fabrics from used garments using ionic liquids [76]. After dissolution, the synthetic polymer could be collected and then recycled, while the extracted cellulose and keratin could be regenerated by wet spinning. It was also found that the ionic liquids could be recovered and reused repeatedly by distillation, creating a complete closed-loop system for textile recycling.

Similarly, the use of ionic liquids to recycle hemp fabrics has yielded intriguing results [77]. In this approach, the hemp fabrics were subjected to a pretreatment with a sulfuric acid solution, with the objective of adjusting the viscosity, and then dissolved in 1,5-diazabicyclo[4.3.0]non-5-enium acetate. The resulting pulp was regenerated by dry-jet wet spinning, and the regenerated fibers were spun into yarn and knitted into a fabric. In particular, the mechanical analysis showed that the fabrics made with regenerated fibers had a good modulus of elasticity and improved abrasion resistance compared to the original hemp fabric. In the same way, Ma and colleagues investigated the dissolution of cotton from denim waste [71]. In this case, the process involved the use of an ionic liquid and DMSO as a cosolvent. The addition of the cosolvent facilitated the rapid dissolution of the cotton, thereby improving the spinnability of the resulting pulp. The mechanical test showed that the mechanical properties were adequate and comparable to those observed for traditional viscose fibers.

**Figure 7:**
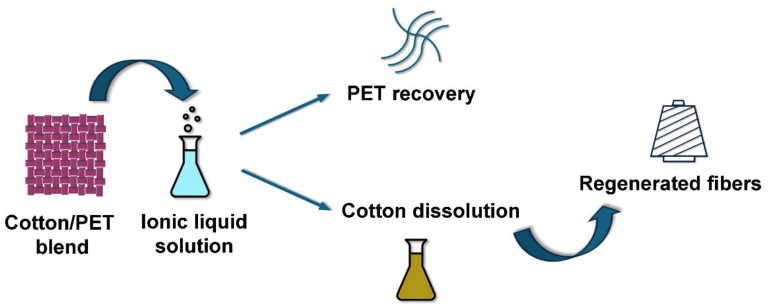
Schematic representation of cotton/PET separation and recovery.

Following the recovery of cellulose from textile waste using ionic liquids, a recent study explored a promising new method for regenerating short wool fibers [75]. These fibers, which cannot normally be spun, have been dissolved using 1-allyl-3-methylimidazolium dicyanamide at 130 °C in the presence of a suitable reducing agent such as mercaptoethanol. In particular, the dissolution of wool results in the formation of a soluble fraction containing low molecular weight polypeptide chains and an insoluble fraction that can be further processed. Another interesting study involves the separation of natural fibers such as wool and cotton from polyester [76]. In this study, Sun and colleagues attempted to recycle fast fashion products by separating the cotton/polyester and wool/polyester blends using 1-butyl-3-methylimidazolium chloride and 1,3-dimethylimidazolium dimethyl phosphate. As in the previous case, the undissolved solid polyester was easily removed, while the extracted cellulose and wool keratin could be regenerated by wet spinning. A schematic representation of the process for cotton is given in Figure 7.

In contrast, other studies have looked at the potential use of ionic liquids in open-loop systems to convert textile waste into high-value-added products. For example, Arshi et al. investigated the importance of pretreating cotton waste for the production of bioethanol [80]. Specifically, the cotton waste was treated with ionic liquids instead of the conventional acid pretreatment with phosphoric acid. It appears that the application of an ionic liquid at 175 °C facilitates the conversion of cotton waste to bioethanol via a process of simultaneous saccharification and fermentation. This represents a promising strategy for utilizing cotton waste textiles as a source of biomass for the future production of renewable biofuels. Similarly, Hong et al. investigated the potential of cotton-based waste textiles as an alternative feedstock for bacterial cellulose production [81]. In this case, the cellulosic fabrics were subjected to pretreatment with 1-allyl-3-methylimidazolium chloride and subsequently processed through enzymatic saccharification. The pretreated fabrics demonstrated a five- to sevenfold increase in the rate of enzymatic hydrolysis and produced a sevenfold greater yield of fermentable sugars in comparison to the unprocessed fabrics. Furthermore, the resulting bacterial cellulose exhibited a yield of 10.8 g/L, representing an 83% enhancement in productivity when compared to that of the culture grown in a glucose-based medium.

### 3.4. Dyed Textiles and Wastewater Treatment

**Figure 8:**
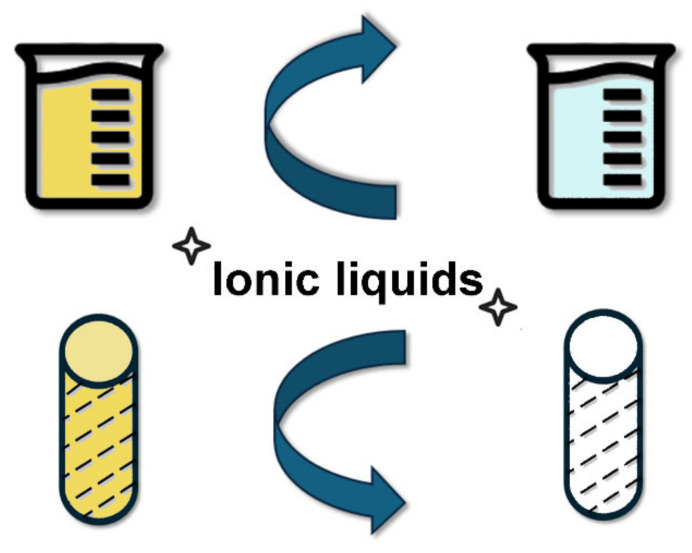
Schematic representation of the use of ionic liquids in dyed textile and wastewater treatment. In particular, the figure illustrates the dual role of ionic liquids as effective extraction agents for dye removal from wastewater and textiles. Key mechanisms include dye adsorption, solubilization, and ionic exchange. Applications depicted range from the removal of acid dyes and reactive dyes from aqueous solutions to the recovery of colorants and reuse of treated textiles. The process highlights the potential of ILs for sustainable wastewater treatment and recycling in the textile industry.

Textile wastewater is a major environmental concern, as the industry generates large volumes of polluted water containing toxic chemicals, dyes, and heavy metals [82]. This wastewater is often difficult to treat and, when discharged into natural water bodies, can damage aquatic ecosystems, contaminate drinking water sources, and affect human health [83]. It is, therefore, imperative to develop effective methods for the treatment of dye effluent in order to prevent the adverse ecological impacts associated with its direct release into the environment. In recent years, a number of studies have demonstrated the efficacy of ionic liquids in the removal of textile dyes from aqueous solutions and textile products, as summarized in Figure 8.

For example, Vijayaraghavan et al. successfully removed CI Acid Blue 113 and CI Acid Red 115 from water with an extraction efficiency of 98% using 1-butyl-1-methylpyrrolidinium bis(trifluoromethylsulfonyl)imide [84]. Concurrently, Li et al. explored the extraction of CI Acid Yellow 25 and CI Acid Red 14 from aqueous solutions using 1-butyl-3-methylimidazolium hexafluorophosphate, achieving extraction yields of 60–99% for these dyes [85,86]. A similar study showed that the use of imidazolium-based ionic liquids is an effective method for the removal of acid dyes, especially when the pH is carefully regulated. Under optimized pH conditions, the extraction efficiency ranged from 52% to 100%, depending on the type of acid dye [87]. As an additional consideration, Fan et al. have demonstrated that the efficacy of dye removal by ionic liquids is closely correlated with the length of the alkyl chain on the cations [88].

Another approach is based on the synthesis or modification of some known absorbents using ionic liquids to improve their performance. For example, Fat’hi et al. stabilized 1-butyl-3-methylimidazolium hexafluorophosphate on alumina to increase the absorbent’s capacity to remove indigo carmine [89]. It has been reported that, under optimized conditions, removal efficiencies of more than 95% can be achieved. In other studies, 1-methyl-3-(triethoxysilylpropyl)imidazolium and 1-propyl-3-methylimidazolium chloride were grafted onto nanoporous silica [90,91]. In both cases, the silica properties were improved in acid dye removal. Later, Lawal et al. reported the adsorption of C.I. Acid Red 1 on kaolin modified with 1-hexyl-3-decahexylimidazolium bromide. They showed that this modified kaolin offers a high adsorption capacity [92]. Moreover, amino ionic liquid modified super paramagnetic mesoporous core/shell nanocomposite and its application as adsorbent to remove two azo acid dyes has been explored [93]. In a recent study, Thasneema et al. investigated the potential of phosphonium-based room-temperature ionic liquids for the extraction of toxic compounds, including textile dyes such as Rhodamine B, Methylene Blue, Methyl Orange, Malachite Green, Alizarin Red S, and Congo Red dyes, from their aqueous solution [24]. The results showed that this class of ionic liquids exhibited remarkable removal capabilities by adjusting concentration, pH, and contact time.

However, the potential for the functionalization of ionic liquids was also investigated. For example, functionalized ionic liquids were synthesized through a cross-linking reaction between 1-aminoethyl-3-vinylimidazolium chloride and divinylbenzene. The functionalized ionic liquids were shown to selectively remove acid dyes selectively [94]. In a similar approach, Kamran et al. investigated the modification of Fe_3_O_4_ using 1-octyl-3-methylimidazolium bromide and its potential to remove several reactive dyes [95]. As evidenced by their experimental results, the modified Fe_3_O_4_ nanoparticles showed remarkable efficacy in the removal of reactive dyes, with a removal efficiency of over 98% observed.

Ionic liquids have also been shown to be effective in removing dyes from textiles. Chen et al. investigated the potential use of nonfluorine quaternary ammonium-based ionic liquids for the removal of acid dyes. Among the ionic liquids investigated, tricaprylmethylammonium thiocyanate showed the optimal ability to extract acid dyes, with an extraction efficiency of 89% [96]. In a recent study, Mu and colleagues demonstrated that precise control of fiber density could be achieved by using a combination of water, glycerol, dimethylsulfoxide (DMSO), tetramethylurea, ethylene carbonate, isopropyl alcohol and 1,4-butanediol to remove dyes from fabrics effectively. The results showed that the process was able to remove 100% of dispersed dyes, acid, and direct dyes from PET, nylon 6,6, and cotton effectively. In addition, the molecular weight of the polymers remaining after dye removal was found to be almost identical [97]. Another interesting approach is the use of no-ionic surfactants, such as Triton X in the removal of CI Basic Blue 9 with 1-butyl-3-methylimidazolium hexafluorophosphate [98]. The inclusion of the nonionic surfactant was found to increase the extraction efficiency to 97.8%.

## 4. Benefits of Ionic Liquids in Textile Applications

**Figure 9:**
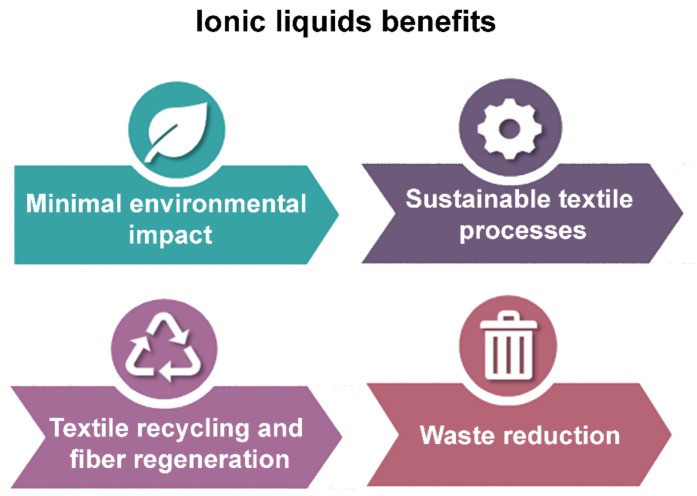
Benefits associated with the use of ionic liquids in the textile sector.

As shown in the previous paragraphs, ionic liquids are increasingly being used in the textile industry. Their success is due not only to their intrinsic properties but also to a number of additional benefits. For example, the use of ionic liquids is considered to have a minimal impact on the environment, as they facilitate sustainability throughout the various stages of textile production, including fiber dissolution, dyeing, and recycling [24]. Firstly, unlike traditional solvents, the negligible volatility of ionic liquids significantly reduces the emission of volatile organic compounds (VOCs), which are known to contribute to air pollution and associated health risks [99]. In textile processing and recycling, ionic liquids enable the efficient dissolution and regeneration of some fibers, such as cellulose, facilitating recycling processes that use less energy and water than conventional mechanical or chemical methods [71,75,80]. Some of the advantages of ionic liquids are summarized in Figure 9.

Furthermore, one of the critical advantages of ionic liquids is their potential for recovery and reuse, which is particularly important in ensuring the environmental and economic sustainability of textile applications. Due to their negligible vapor pressure and high thermal stability, ionic liquids can often be recovered through distillation, liquid-liquid extraction, or adsorption techniques [100]. During textile applications such as dyeing and finishing, spent ionic liquids can be separated from wastewater using processes like phase separation or membrane filtration, as used for other applications [101]. For instance, the ionic liquid can be precipitated by altering the solution’s pH or by adding a secondary solvent that induces phase separation. This allows for efficient recovery with minimal loss of material. Recycled ionic liquids typically retain their functional properties, enabling multiple cycles of reuse in processes like modification [76]. However, challenges such as contamination with impurities or partial degradation may arise. The integration of closed-loop systems utilizing ionic liquids has the potential to significantly reduce waste generation and environmental impact, aligning with circular economy principles in the textile industry [76].

In addition, ionic liquids offer significant advantages in textile dyeing, a process typically associated with large volumes of water and chemical waste [24]. Their tunable solubility and ability to act as selective solvents allow dyes to be incorporated into fibers, reducing the need for auxiliary chemicals. This not only minimizes the volume of wastewater generated but also reduces the concentration of residual dyes in the effluent, thereby reducing the environmental impact of textile dyeing operations [102]. The biodegradability and recyclability of many ionic liquids further enhance their environmental profile. Unlike traditional solvents, which can persist in the environment, most ionic liquids can be recovered and reused, minimizing waste generation [100]. However, some research has also shown that ionic liquids can support innovative textile treatments, such as antimicrobial or flame retardant properties, without relying on hazardous substances, thereby promoting safer and more sustainable product development [103]. Taken together, these benefits highlight the potential of ionic liquids not only to reduce the environmental impact of the textile industry but also to drive the transition to a circular economy by enabling closed-loop processes and more efficient resource use.

## 5. Ecological Concerns of Ionic Liquids

While ionic liquids have gained significant attention for their unique properties, their environmental impact raises some concerns. Their persistence, toxicity, and limited biodegradability pose ecological risks that must be carefully evaluated [104]. For example, it has been demonstrated that the ecological effects of ionic liquids are influenced by their chemical structure [105]. The cation type, side-chain length, and hydrophobicity play major roles in toxicity, while the anion typically contributes to a lesser extent. Furthermore, ionic liquids are often poorly biodegradable, making them persistent pollutants in the environment. For instance, they exhibit resistance to microbial degradation, and even when degradation occurs, intermediates may still exhibit toxicity. This underscores the need for further research into degradation pathways [11,104]. In the end, there is a possibility that ionic liquids can be discharged into aquatic or terrestrial ecosystems. ILs have the potential to persist for extended periods, accumulating in soil and water bodies. Their environmental degradation pathways are not well understood, and studies suggest that their biodegradability depends heavily on the specific cation–anion combination [104,106,107].

## 6. Future Research Directions

Ionic liquids have attracted considerable attention in the textile industry due to their unique properties, which make them ideal for various applications such as dyeing, textile finishing and fiber recycling. For example, their low vapor pressure reduces the emission of harmful volatile organic compounds (VOCs) during processing, making them a more environmentally friendly alternative to conventional solvents. In addition, their ability to dissolve both organic and inorganic compounds, including natural fibers such as cellulose, opens up opportunities for more sustainable textile production methods. Despite these advantages, the textile industry has not yet fully embraced ionic liquids for widespread use.

**Figure 10:**
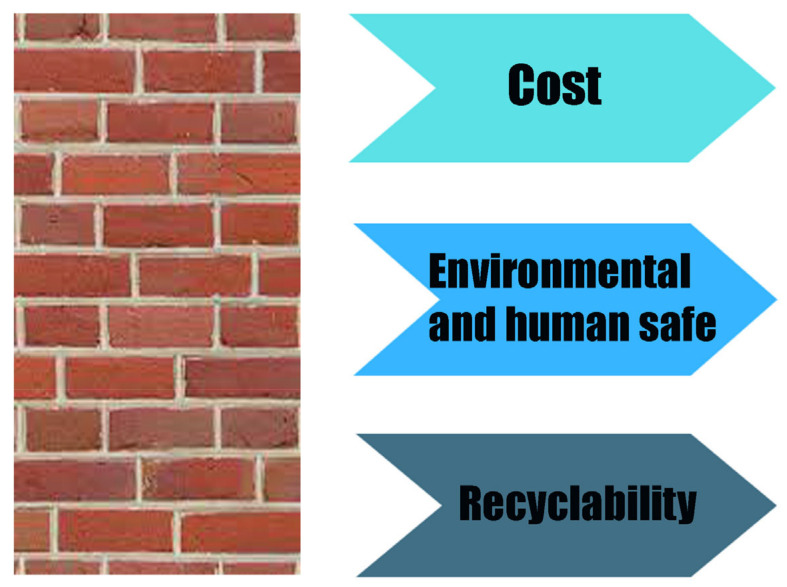
Potential obstacles to the use of ionic liquids in the textile industry.

Although research into the use of ionic liquids to recycle natural fibers such as cotton or wool has shown positive results, further research is needed to make these methods more efficient and commercially viable on a larger scale. However, while there has been considerable progress in the use of ionic liquids for natural fibers, synthetic fibers such as polyester present a greater challenge. Preliminary studies on PET recycling have used both deep eutectic solvents based on choline chloride and imidazole-based ionic liquids, but further research is essential to fully develop efficient recycling methods for synthetic fibers [108]. In addition, further efforts are being made to ensure that ionic liquids are fully exploited in the textile sector. While many ionic liquids are currently synthesized from traditional chemical compounds, there is growing interest in creating biobased ionic liquids. These would be derived from renewable resources and designed to be nontoxic and biodegradable, reducing the environmental impact of their use in textiles. Further research is needed to develop these biobased variants, optimize their performance in textile processes and ensure that they are safe for both humans and the environment [109]. Finally, a significant barrier to the widespread use of ionic liquids in textiles is their cost. Ionic liquids are generally more expensive to produce than conventional solvents, which limits their appeal for large-scale industrial applications [110]. Future research must focus on cost reduction strategies, such as finding cheaper raw materials for ionic liquid production, developing more efficient synthesis processes and improving their recyclability. In addition, the integration of ionic liquids into existing textile processing systems is crucial, as industries are reluctant to overhaul their infrastructure for new technologies unless the benefits (cost savings, environmental, etc.) are clear and significant. All these barriers are summarized in Figure 10.

This breakdown shows that while ionic liquids hold great promise for the textile industry, particularly in recycling, dyeing and sustainable fiber production, significant research is still needed to optimize their performance, reduce costs and expand their commercial application. The future of ionic liquids in textiles will depend on overcoming these scientific and economic hurdles.

## 7. Conclusions

Ionic liquids have emerged as a promising alternative to conventional solvents in the textile industry, offering a number of advantages, including low volatility, high thermal stability, tunable solubility, and recyclability. Although the use of ionic liquids is still at the laboratory scale, this review highlights their potential applications in dyeing processes, textile finishing, fiber preparation, and textile waste recycling. Despite these considerable advantages, the full potential of ionic liquids has yet to be realized due to a number of challenges, including high production costs, limited scalability, and the need for further integration into existing textile infrastructures. Overcoming these barriers will not only stimulate innovation within the textile industry but will also make a significant contribution to the transition to a circular economy, where waste is minimized and resources are efficiently reused. The future of ionic liquids in textiles is promising, but it depends on continued research efforts to realize their potential fully.

## Figures and Tables

**Table 1:** Various applications of ionic liquids in textile dyeing processes.

Dye Type	Type of Material	Ionic Liquids	Dyeing Process	Process Condition	Role of Ionic Liquid	Reference
Acid dyes	Wool	Methylimidazolium-based ionic liquids	Exhaustion	60 °C, 60 min	Pretreatment	[37]
		Diethylethanolamine-based ionic liquids				
	Polyamide 6.6	Methylimidazolium-based ionic liquids	Exhaustion	80 °C, 45 min	Dyeing medium	[38]
		Diethylethanolamine-based ionic liquids				
	Silk	Methylimidazolium-based ionic liquids	Exhaustion	60 °C, 30 min	Pretreatment	[39]
	Cotton	Diethylethanolamine-based ionic liquids	/	/	Dyeing medium	[40]
	Polyester	Diethylethanolamine-based ionic liquids	/	/	Dyeing medium	[40]
	Polyacrylonitrile	Diethylethanolamine-based ionic liquids	/	/	Dyeing medium	[40]
		Imidazole-based ionic liquids				
	m-aramid	Methylimidazolium-based ionic liquids	Exhaustion	180 °C, 60 min	Dyeing medium	[41]
Reactive dyes	Wool	Methylimidazolium-based ionic liquids	Exhaustion	80 °C, 60 min	Pretreatment	[39]
	Silk	Methylimidazolium-based ionic liquids	Exhaustion	80 °C, 60 min	Pretreatment	[39]
	Cotton	Quaternary ammonium salt ionic liquids	Pad-dry-cure	60 °C, 10–20 min	Auxiliary	[36]
	Flax	Methylimidazolium-based ionic liquids	/	40–80 °C, 150 min	Auxiliary	[42]
Basic dyes	Polyacrylonitrile	Methylimidazolium-based ionic liquids	Exhaustion	85 °C, 50 min	Retardant	[43]
Disperse dyes	Polyester	Methylimidazolium-based ionic liquids	Open-vessel	95 °C	Additives	[35,44]
	Wool	Methylimidazolium-based ionic liquids	Open-vessel	95 °C	Additives	[35]
	Cotton	Methylimidazolium-based ionic liquids	Open-vessel	95 °C	Additives	[35]
Direct dyes	Cotton	Methylimidazolium-based ionic liquids	Impregnation	50 °C, 30 min	Dyeing medium	[44]

**Table 2:** Summary of ionic liquid applications in textile finishing.

Application	Material	Ionic Liquids	Process	Outcome	Reference
Antimicrobial properties	Wool	Imidazolium-based ionic liquids	Pretreatment with ionic liquids to bind keratin.	Longlasting effect against *E. coli*.	[59]
	Viscose	Imidazolium-based ionic liquids	Treated with anionic agents followed by ionic liquids.	Antibacterial resistance against Gram-positive and Gram-negative bacteria.	[60]
Flame retardancy	Cotton	Methylimidazolium and pyridinium-based ionic liquids	Coating applied via sol-gel method.	Improved LOI (limiting oxygen index) and flame retardancy.	[61]
	Cotton	Various (e.g., PF_6_^−^)	Ionic liquid applied in finishing bath.	Enhanced flame retardant and water repellency properties.	[62]
UV protection	Cotton	1-Butyl-3-methylimidazolium chloride + nano-ZnO	Nano-zinc oxide applied to cotton fabric using ionic liquids.	Excellent UV protection and bacteriostatic activity.	[63]
Surface Tactility	Polyester	Cetyltrimethylammonium bromide and 1-butyl-3-methylimidazolium chloride	Deposition of cellulosic materials on polyester fibers.	Imparts a cotton-like feel to synthetic fibers.	[58]

**Table 3:** Ionic liquids used in textile recycling.

Material Recycled	Type of Ionic Liquids	Reference
Cotton	1-allyl-3-methylimidazolium chloride	[70]
	1,5-diazabicyclo[4.3.0]non-5-enium acetate	[57]
	1-butyl-3-methylimidazolium acetate and DMSO as a cosolvent.	[71]
Cotton/PET	Superbase-based ionic liquid	[72]
	1-allyl-3-Methylimidazolium chloride	[73]
	1-ethyl-3-methylimidazolium diethyl phosphate DMSO system	[74]
	1-allyl-3-methylimidazole chloride in conjunction with DMSO	[74]
	1-allyl-3-methylimidazolium dicyanamide	[75]
Wool	1-allyl-3-methylimidazolium dicyanamide	[75]
Wool/Polyester	1,3-dimethylimidazolium dimethyl phosphate	[76]
Hemp	1,5-diazabicyclo[4.3.0]non-5-enium acetate	[77]
